# Relationship between uric acid to high-density lipoprotein cholesterol ratio and sarcopenia in NHANES: exploring the mediating role of bilirubin and association with all-cause mortality

**DOI:** 10.3389/fnut.2025.1560617

**Published:** 2025-06-23

**Authors:** Haiyan Mao, Xiaoying Zhang, Shanshan Huang, Tong Lin, Zhikui Chen

**Affiliations:** ^1^Department of Critical Care Medicine, Ningbo Medical Center Lihuili Hospital, Ningbo, China; ^2^Department of Geriatrics, Ningbo Medical Center Lihuili Hospital, Ningbo, China; ^3^Department of Cardiovascular Medicine, Ningbo Medical Center Lihuili Hospital, Ningbo, China

**Keywords:** uric acid to high-density lipoprotein cholesterol ratio, sarcopenia, bilirubin, oxidative stress, all-cause mortality, NHANES

## Abstract

**Background:**

Sarcopenia is a systemic disease characterized by a decline in muscle mass and function. It is associated with adverse health outcomes, and younger patients are at higher risk. Thus, early identification and prevention of high-risk factors are crucial. The uric acid to high-density lipoprotein ratio (UHR) is a novel marker of inflammation and metabolism, but studies on its association with sarcopenia are currently lacking.

**Methods:**

Data from the National Health and Nutrition Examination Survey (NHANES) 2011–2018 were utilized. Weighted multivariate logistic regression analysis was performed to explore the association between UHR and sarcopenia. Causal mediation analysis was conducted to investigate the mediating role of oxidative stress factors and systemic inflammatory markers in the UHR-sarcopenia relationship. Subgroup analysis and interaction tests were performed to identify high-risk populations for the positive association between UHR and sarcopenia. Restricted cubic spline (RCS) explored potential non-linear relationships between UHR and sarcopenia. Weighted multivariate Cox proportional hazards regression analysis assessed the relationship between UHR and all-cause mortality in sarcopenia patients.

**Results:**

A total of 10,308 adult participants aged ≥ 20 years were included in the study, with 901 diagnosed with sarcopenia. The weighted multivariate logistic regression analysis showed a significant positive association between UHR and sarcopenia after adjusting for all confounding factors (OR = 1.057; 95% CI: 1.037–1.077; *P* < 0.001). Total bilirubin mediated −8.53% of the association between UHR and sarcopenia (95% CI: −13.42% to −5.91%; *P* < 0.001). The subgroup analysis and interaction test results indicate that the positive association between the two variables is relatively stable across different populations. RCS analysis revealed no significant non-linear relationship between UHR and sarcopenia (*P* = 0.167). Weighted multivariate Cox proportional hazards regression analysis showed a significant positive association between UHR and all-cause mortality in sarcopenia patients (HR = 1.053; 95% CI: 1.024–1.083; *P* < 0.001) in the unadjusted model. However, after adjusting for all covariates, UHR maintained a positive association with all-cause mortality in sarcopenia patients (HR = 1.023; 95% CI: 0.990–1.056), though this association did not reach statistical significance (*P* = 0.173).

**Conclusion:**

Elevated UHR shows a significant association with sarcopenia prevalence and exhibits a positive association trend with all-cause mortality among affected individuals. These findings suggest that UHR may serve as a potential indicator for sarcopenia risk assessment. Further prospective studies are warranted to validate its clinical utility for early screening and intervention strategies.

## Introduction

Sarcopenia is an age-related chronic disease characterized by progressive loss of skeletal muscle mass and strength, as well as a decline in physical function (1). It increases the risk of falls, frailty, osteoporosis, fractures, metabolic disorders (such as type 2 diabetes and cardiovascular diseases), and even mortality (2). As global populations age, the incidence of sarcopenia has been rising annually. It is estimated that the global prevalence of sarcopenia ranges from 10% to 27%, with even higher rates among individuals over 80 years old (1, 3).

The causes of sarcopenia include malnutrition, physical inactivity, endocrine changes, chronic diseases (such as cancer and cardiovascular diseases), and genetic factors (4). Its pathogenesis is complex, with inflammation and oxidative stress key mechanisms (5). The Systemic Immune-Inflammation Index (SII), neutrophil-to-lymphocyte ratio (NLR), and platelet-to-lymphocyte ratio (PLR) are biomarkers of systemic inflammation (6), and they are rapid and critical indicators for evaluating subclinical inflammation in various diseases (7, 8). However, in addition to inflammation, oxidative stress represents another key pathogenic mechanism in sarcopenia. Bilirubin, as a potent endogenous antioxidant, mitigates oxidative damage by scavenging free radicals and suppressing inflammatory response processes that are closely implicated in the pathogenesis of various metabolic disorders (e.g., diabetes, non-alcoholic fatty liver disease, and cardiovascular diseases) (9–11). Notably, existing studies have demonstrated that lower serum bilirubin levels are significantly associated with an increased risk of sarcopenia (12).

Uric acid, a product of purine metabolism, is closely linked to inflammation and oxidative stress (13). High-density lipoprotein cholesterol (HDL-C) has specific anti-inflammatory and antioxidant effects and is related to the pathogenesis of insulin resistance and metabolic syndrome (14–16). The uric acid to high-density lipoprotein cholesterol ratio (UHR) is a new inflammatory and metabolic marker that has been associated with increased risks of metabolic syndrome, abdominal aortic aneurysm, non-alcoholic fatty liver disease, and cardiovascular diseases (17–22).

Sarcopenia, a progressive muscle-wasting syndrome associated with multiple chronic diseases, significantly impairs quality of life in older adults, with younger patients facing even higher risks of adverse health outcomes (1, 23–25). Given the lack of specific clinical manifestations and targeted therapies, early identification of modifiable risk factors is critical for implementing effective interventions. UHR, a composite index integrating pro-oxidant (uric acid) and antioxidant (HDL-C) components (17), provides a comprehensive assessment of systemic oxidative stress. This routinely measurable biomarker offers practical advantages for clinical translation, including operational simplicity, cost-effectiveness, and high reproducibility. Notably, the association between UHR and sarcopenia remains unexplored, particularly regarding its potential mediation through inflammatory and oxidative stress pathways. Investigating UHR’s predictive value thus holds significant theoretical and clinical implications. Therefore, this study aims to utilize data from the National Health and Nutrition Examination Survey (NHANES), a nationwide survey initiated by the National Center for Health Statistics (NCHS) under the United States Centers for Disease Control and Prevention (CDC), which measures the health and nutritional status of United States adults and children. The study will explore the relationship between UHR and the risk of sarcopenia, investigate the mediating role of inflammation and oxidative stress in the UHR-sarcopenia association, and examine the relationship between UHR and all-cause mortality in sarcopenia patients.

## Materials and methods

### Study design and population

**FIGURE 1:**
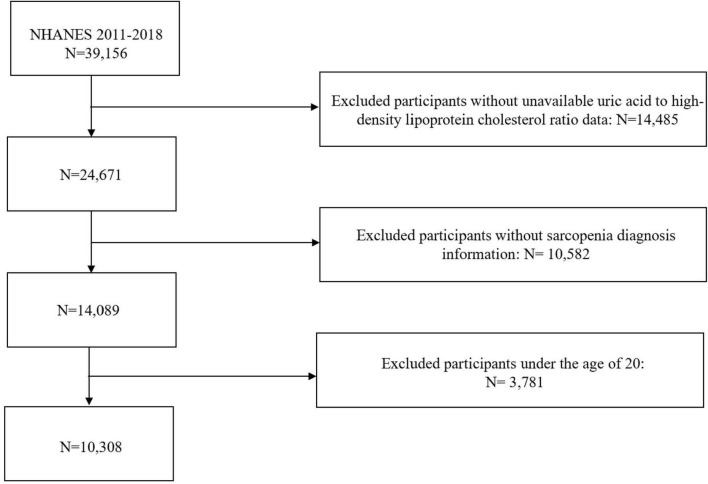
Screening conditions and process for the study population.

This study utilized data from four cycles of NHANES (2011–2018)^1^. NHANES employs a multistage sampling method to ensure broad population representativeness. Participants were excluded if they were under 20 years of age or had missing data on UHR or sarcopenia diagnosis. The detailed study process is illustrated in Figure 1.

### Diagnosis of sarcopenia

Sarcopenia is assessed by the sum of appendicular lean mass (ALM). ALM is measured using Dual Energy X-ray Absorptiometry (DEXA), which is the primary method for measuring body composition (26). Participants who are pregnant, weigh more than 136.4 kg, are taller than 192.5 cm, have a history of using radiographic contrast agents (such as barium) within the past 7 days, or have undergone nuclear medicine studies in the past 3 days were excluded from the study. The diagnosis of sarcopenia is based on ALM divided by body mass index (BMI), with cutoff values of < 0.789 for males and < 0.512 for females (27).

### Measurement of UHR

The formula to calculate the UHR (%) is as follows: UHR = Serum uric acid (mg/dL)/HDL-C (mg/dL) × 100% (22). Blood samples were collected by trained professionals at the Mobile Examination Center (MEC). Serum uric acid (SUA) was measured using the timed endpoint method, while HDL-C concentration was measured by the enzymatic method (28). Detailed experimental methods can be found on the NHANES website.

### Measurement of oxidative stress factor (total bilirubin) and systemic inflammatory markers (SII, PLR, NLR)

The Beckman Coulter DXH 800 instrument was used to measure blood cell counts, including monocytes, neutrophils, lymphocytes, and platelets. SII was calculated as: SII = (platelet count × neutrophil count)/lymphocyte count (6, 29). PLR was calculated as: PLR = platelet count/lymphocyte count, and NLR was calculated as: NLR = absolute neutrophil count/absolute lymphocyte count (8). Total bilirubin concentration was measured using the Beckman Coulter UniCel DxC800 instrument (Brea, CA), employing a timed-endpoint Diazo method (Jendrassik–Grof method) (30).

### Other covariates

We automatically included fundamental demographic variables (gender, age, race/ethnicity, poverty income ratio (PIR), and education level) as essential confounders based on their established role as potential effect modifiers. Additional covariates were systematically selected through comprehensive review of documented sarcopenia risk factors in the literature (4, 5), encompassing laboratory indicators like albumin, alanine aminotransferase (ALT), aspartate aminotransferase (AST), estimated glomerular filtration rate (eGFR), smoking status, drinking history, and comorbid conditions (such as coronary artery disease, stroke, cancer history), physical activity level, and sedentary time. Smoking status, drinking history, physical activity, sedentary time, and comorbidity data were collected via questionnaires. The levels of physical activity encompassed leisure activities, activities related to work, and transport activities. The total physical activity level is calculated by summing these three types of activities, with leisure and work-related activities determined by adding twice the duration of high-intensity activities to the moderate-intensity activities (31). As per the 2018 United States Physical Activity Guidelines, physical activity was divided into these categories: 0, 1–149, 150–299 min weekly, and 300 or more minutes weekly. Sedentary behavior was categorized as: 0–179, 180–299, 300–419, and ≥ 420 min daily (32). The CKD-EPI Creatinine Equation (2021) was used to calculate eGFR for evaluating kidney function (33).

### Mortality outcome

Mortality records from the National Death Index (NDI), managed by the CDC, were connected with NHANES data. The NDI offers comprehensive death data across the country, detailing causes, dates, and other pertinent information. The determination of death status was achieved by probabilistically matching with NDI records using a unique research identifier for deaths that occurred before 31 December 2019. Detailed matching methods can be obtained from the National Center for Health Statistics. Deaths were categorized using ICD-10 codes, with the main focus of the study being mortality from all causes (Codes I00-I09) (34).

### Statistical analyses

Following NHANES guidelines and recommended weights, statistical analysis was conducted, utilizing the Kolmogorov-Smirnov test to check for data normality. The median and interquartile range (IQR) were applied to non-normally distributed continuous variables, with group comparisons performed via the Mann-Whitney U test. Proportions were used to express categorical variables, and the Chi-square test was employed for group comparisons. Weighted multivariate logistic regression was employed to examine the relationship between UHR and sarcopenia. Three models were created: Model 1 (no adjustments), Model 2 (adjusted for gender, age, race, education level, and PIR), and Model 3 [adjusted for all covariates except potential mediators (total bilirubin, SII, PLR, and NLR)]. Subgroup and interaction tests were conducted to assess whether the relationship between the variables remains robust in specific high-risk populations. A restricted cubic spline (RCS) was used to explore the potential non-linear relationship between UHR and sarcopenia.

Mediation analysis was conducted using parallel mediation analysis (R package “mediation”) with 1,000 bootstrap samples to assess the potential mediating effects of oxidative stress factors (total bilirubin) and systemic inflammation markers (SII, PLR, NLR) on the UHR-sarcopenia association. The direct effect (DE) represents the impact of UHR on sarcopenia without mediation by total bilirubin or systemic inflammation markers (SII, PLR, NLR). The indirect effect (IE) reflects the mediation of total bilirubin or systemic inflammation markers (SII, PLR, NLR) in the UHR-sarcopenia relationship. The mediation effect proportion was calculated by dividing the IE by the total effect (TE).

Weighted multivariate Cox proportional hazards regression was used to assess the association between UHR and all-cause mortality in sarcopenia patients. Similar to the logistic regression models, three models were constructed: Model 1 (unadjusted), Model 2 (adjusted for gender, age, race, education level, and PIR), and Model 3 [adjusted for all covariates except potential mediators (total bilirubin, SII, PLR, and NLR)].

All statistical analyses were performed using R software (version 4.0.0) and SPSS (version 25.0), with a significance level set at *P* < 0.05.

## Results

### Baseline characteristics of the study population

Table 1 presents the baseline characteristics of the study population. A total of 10,308 adults aged ≥ 20 years were included in this study, with 901 diagnosed with sarcopenia. When stratified by UHR quartiles, a clear dose-response relationship emerged, with sarcopenia prevalence progressively increasing from 5.62% in Q1 to 12.11% in Q4. Participants in the highest UHR quartile (Q4) exhibited distinct clinical characteristics compared to Q1, including significantly higher proportions of males (81.17% vs. 15.82%), greater prevalence of hypertension (31.08% vs. 16.92%), diabetes (9.75% vs. 4.50%), and current smoking (45.18% vs. 33.48%), along with more adverse metabolic profiles featuring elevated ALT, AST, and uric acid levels coupled with lower HDL-C and eGFR (all *P* < 0.05).

**TABLE 1:** Baseline characteristics of the study population.

Variables	Total (*n* = 10,308)	Q1 (*n* = 2,579)	Q2 (*n* = 2,576)	Q3 (*n* = 2,577)	Q4 (*n* = 2,576)	*P-*value
**Sarcopenia, *n* (%)**						< 0.001
Yes	901 (8.74)	145 (5.62)	215 (8.35)	229 (8.89)	312 (12.11)	–
No	9407 (91.26)	2434 (94.38)	2361 (91.65)	2348 (91.11)	2264 (87.89)	–
Age, years, mean (Q_1_, Q_3_)	39.00 (29.00, 49.00)	40.00 (30.00, 49.00)	39.00 (28.00, 50.00)	40.00 (29.00, 49.00)	39.00 (30.00, 49.00)	0.51
**Age group, *n* (%)**						0.448
< 40	5188 (50.33)	1271 (49.28)	1319 (51.20)	1284 (49.83)	1314 (51.01)	–
≥ 40	5120 (49.67)	1308 (50.72)	1257 (48.80)	1293 (50.17)	1262 (48.99)	–
**Gender, *n* (%)**						< 0.001
Male	5078 (49.26)	408 (15.82)	977 (37.93)	1602 (62.17)	2091 (81.17)	–
Female	5230 (50.74)	2171 (84.18)	1599 (62.07)	975 (37.83)	485 (18.83)	–
**Race, *n* (%)**						< 0.001
Mexican American	1562 (15.15)	330 (12.80)	407 (15.80)	411 (15.95)	414 (16.07)	–
Other Hispanic	1082 (10.50)	279 (10.82)	265 (10.29)	267 (10.36)	271 (10.52)	–
Non-Hispanic White	3598 (34.90)	913 (35.40)	845 (32.80)	907 (35.20)	933 (36.22)	–
Non-Hispanic Black	2121 (20.58)	592 (22.95)	576 (22.36)	507 (19.67)	446 (17.31)	–
Other race/multiracial	1945 (18.87)	465 (18.03)	483 (18.75)	485 (18.82)	512 (19.88)	–
**Education level, *n* (%)**						< 0.001
Less than 9th grade	644 (6.25)	131 (5.08)	164 (6.37)	161 (6.25)	188 (7.30)	–
9–11th grade	1226 (11.90)	256 (9.93)	310 (12.04)	306 (11.87)	354 (13.74)	–
High school grade/GED or equivalent	2244 (21.77)	472 (18.31)	569 (22.10)	601 (23.32)	602 (23.37)	–
Some college or AA degree	3389 (32.88)	853 (33.09)	843 (32.74)	866 (33.60)	827 (32.10)	–
College Graduate or above	2803 (27.20)	866 (33.59)	689 (26.76)	643 (24.95)	605 (23.49)	–
PIR, mean (Q_1_, Q_3_)	2.14 (1.07, 4.12)	2.46 (1.15, 4.58)	2.11 (1.06, 3.92)	2.14 (1.06, 4.12)	1.98 (1.05, 3.82)	< 0.001
Total cholesterol, mg/dL, mean (Q_1_, Q_3_)	187.00 (163.00, 214.00)	186.00 (164.00, 211.50)	184.00 (161.00, 210.00)	188.00 (162.00, 215.00)	191.00 (164.00, 218.00)	< 0.001
Albumin, g/L, mean (Q_1_, Q_3_)	43.00 (41.00, 45.00)	43.00 (40.00, 45.00)	43.00 (40.00, 45.00)	43.00 (41.00, 46.00)	43.00 (41.00, 45.00)	< 0.001
ALT, U/L, mean (Q_1_, Q_3_)	21.00 (16.00, 29.00)	17.00 (13.00, 21.50)	19.00 (15.00, 25.00)	22.00 (17.00, 30.00)	28.00 (21.00, 40.00)	< 0.001
AST, U/L, mean (Q_1_, Q_3_)	22.00 (18.00, 27.00)	20.00 (17.00, 24.00)	21.00 (18.00, 25.00)	22.00 (19.00, 27.00)	25.00 (20.00, 30.00)	< 0.001
eGFR, mL/min/1.73m^2^, mean (Q_1_, Q_3_)	113.72 (103.77, 125.72)	114.94 (105.00, 130.45)	115.15 (104.61, 128.02)	113.08 (103.60, 124.86)	111.68 (102.45, 121.83)	< 0.001
Total bilirubin, mg/dL, mean (Q_1_, Q_3_)	0.60 (0.40, 0.70)	0.50 (0.40, 0.70)	0.50 (0.40, 0.70)	0.60 (0.40, 0.70)	0.60 (0.40, 0.80)	< 0.001
Uric acid, mg/dL, mean (Q_1_, Q_3_)	5.20 (4.30, 6.20)	3.90 (3.40, 4.50)	4.80 (4.30, 5.40)	5.60 (5.00, 6.20)	6.70 (5.90, 7.40)	< 0.001
HDL-C, mg/dL, mean (Q_1_, Q_3_)	50.00 (41.00, 60.00)	67.00 (59.00, 77.00)	54.00 (48.00, 60.25)	47.00 (42.00, 52.00)	38.00 (34.00, 42.00)	< 0.001
SII, mean (Q_1_, Q_3_)	438.67 (314.27, 611.05)	429.00 (307.40, 611.18)	445.58 (318.61, 630.28)	440.09 (314.30, 605.03)	438.91 (318.89, 600.49)	0.263
NLR, mean (Q_1_, Q_3_)	1.85 (1.41, 2.41)	1.80 (1.38, 2.37)	1.86 (1.41, 2.44)	1.85 (1.43, 2.38)	1.88 (1.43, 2.44)	0.026
PLR, mean (Q_1_, Q_3_)	111.25 (89.05, 138.18)	119.00 (95.00, 147.71)	114.00 (91.60, 141.48)	109.56 (88.02, 133.50)	103.06 (83.23, 129.21)	< 0.001
UHR,%, mean (Q_1_, Q_3_)	10.30 (7.59, 14.08)	6.06 (5.16, 6.84)	8.92 (8.25, 9.59)	11.95 (11.09, 12.86)	17.27 (15.50, 20.00)	< 0.001
**Cancer, *n* (%)**						0.043
Yes	380 (3.69)	118 (4.58)	90 (3.50)	82 (3.18)	90 (3.50)	–
No	9,925 (96.31)	2,460 (95.42)	2,485 (96.50)	2,495 (96.82)	2,485 (96.50)	–
**Stroke, *n* (%)**						0.254
Yes	153 (1.48)	32 (1.24)	39 (1.51)	34 (1.32)	48 (1.86)	–
No	10,152 (98.52)	2,547 (98.76)	2,537 (98.49)	2,541 (98.68)	2,527 (98.14)	–
**Coronary heart disease, *n* (%)**						< 0.001
Yes	101 (0.98)	18 (0.70)	12 (0.47)	27 (1.05)	44 (1.71)	–
No	10,197 (99.02)	2,561 (99.30)	2,563 (99.53)	2,547 (98.95)	2,526 (98.29)	–
**Hypertension, *n* (%)**						< 0.001
Yes	2,419 (23.49)	436 (16.92)	515 (20.00)	669 (25.97)	799 (31.08)	–
No	7,880 (76.51)	2,141 (83.08)	2,060 (80.00)	1,907 (74.03)	1,772 (68.92)	–
**Diabetes, *n* (%)**						< 0.001
Yes	754 (7.32)	116 (4.50)	165 (6.41)	222 (8.61)	251 (9.75)	–
No	9,354 (90.80)	2,433 (94.45)	2,361 (91.69)	2,308 (89.56)	2,252 (87.49)	–
Borderline	194 (1.88)	27 (1.05)	49 (1.90)	47 (1.82)	71 (2.76)	–
**Alcohol use, *n* (%)**						< 0.001
Yes	6,004 (63.38)	1,420 (60.92)	1,428 (60.84)	1,546 (64.69)	1,610 (66.94)	–
No	3,469 (36.62)	911 (39.08)	919 (39.16)	844 (35.31)	795 (33.06)	–
**Smoking status, *n* (%)**						< 0.001
Yes	4,032 (39.13)	863 (33.48)	921 (35.75)	1,085 (42.12)	1,163 (45.18)	–
No	6,272 (60.87)	1,715 (66.52)	1,655 (64.25)	1,491 (57.88)	1,411 (54.82)	–
PA, min/week, mean (Q_1_, Q_3_)	420.00 (75.00, 1360.00)	380.00 (80.00, 1140.00)	420.00 (73.75, 1260.00)	450.00 (75.00, 1500.00)	450.00 (76.00, 1575.00)	< 0.001
**PA, *n* (%)**						0.331
0 min/week	1,957 (19.05)	484 (18.83)	503 (19.59)	482 (18.75)	488 (19.03)	–
1–149 min/week	1,274 (12.40)	325 (12.65)	323 (12.58)	300 (11.67)	326 (12.71)	–
150–299 min/week	1,068 (10.40)	301 (11.71)	263 (10.24)	251 (9.77)	253 (9.86)	–
≥ 300 min/week	5,974 (58.15)	1,460 (56.81)	1,479 (57.59)	1,537 (59.81)	1,498 (58.40)	–
SB,min/d, mean (Q_1_, Q_3_)	360.00 (240.00, 480.00)	360.00 (180.00, 480.00)	360.00 (180.00, 480.00)	360.00 (240.00, 480.00)	360.00 (240.00, 480.00)	0.004
**SB, *n* (%)**						0.066
0–179 min/d	1,420 (13.80)	388 (15.06)	361 (14.04)	351 (13.64)	320 (12.46)	–
180–299 min/d	2,426 (23.58)	575 (22.32)	644 (25.05)	604 (23.47)	603 (23.47)	–
300–419 min/d	2,130 (20.70)	546 (21.20)	508 (19.76)	555 (21.57)	521 (20.28)	–
≥ 420 min/d	4,313 (41.92)	1,067 (41.42)	1,058 (41.15)	1,063 (41.31)	1,125 (43.79)	–

Non-normally distributed continuous variables: median (interquartile range); categorical variables: percentages. Between-group comparisons: Mann-Whitney U test and chi-square tests. Q1–Q4 were grouped into study participants based on the quartile range of UHR level [Q1 (1.35–7.59): the first quartile; Q2 (7.59–10.30): the second quartile; Q3 (10.30–14.08): the third quartile; Q4 (14.09–80.00): the fourth quartile]. SII, systemic immune-inflammation index; NLR, neutrophil-to-lymphocyte ratio; PLR, platelet-to-lymphocyte ratio; eGFR, estimated glomerular filtration rate; UHR, uric acid to high-density lipoprotein cholesterol ratio; HDL-C, high-density lipoprotein cholesterol; PIR, poverty income ratio; ALT, alanine aminotransferase; AST, aspartate aminotransferase; PA, physical activity; SB, sedentary behavior.

### Association between UHR and sarcopenia

Weighted multivariate logistic regression was conducted to explore the association between UHR and sarcopenia. As shown in Table 2, when no covariates were adjusted for (Model 1), for each 1% increase in UHR, the risk of sarcopenia increased by 6.3% (OR = 1.063; 95% CI: 1.048–1.078; *P* < 0.001). After adjusting for gender, age, race, education level, and PIR (Model 2), the significant positive association between UHR and sarcopenia remained (OR = 1.066; 95% CI: 1.048–1.084; *P* < 0.001). Even after adjusting for all covariates (Model 3), the positive association remained significant (OR = 1.057; 95% CI: 1.037–1.077; *P* < 0.001). When UHR was divided into quartiles, with the Q1 group as the reference, the risk of sarcopenia increased with higher UHR levels, with the Q4 group having a 2.874-fold higher risk compared to the Q1 group (OR = 2.874; 95% CI: 2.05–4.029; *P* < 0.001).

**TABLE 2:** Weighted logistic regression analysis of UHR and sarcopenia.

Variables	Model 1		Model 2		Model 3	
	**OR (95% CI)**	***P*-value**	**OR (95% CI)**	***P*-value**	**OR (95% CI)**	***P*-value**
UHR	1.063 (1.048, 1.078)	< 0.001	1.066 (1.048, 1.084)	< 0.001	1.057 (1.037, 1.077)	< 0.001
**Categories**						
Q 1	Reference	–	Reference	–	Reference	–
Q 2	1.767 (1.288, 2.424)	0.001	1.788 (1.27, 2.517)	0.001	1.909 (1.342, 2.715)	0.001
Q 3	1.786 (1.326, 2.405)	< 0.001	1.839 (1.306, 2.59)	0.001	1.784 (1.225, 2.597)	0.003
Q 4	2.959 (2.239, 3.909)	< 0.001	3.099 (2.27, 4.231)	< 0.001	2.874 (2.050, 4.029)	< 0.001
*P* for trend	–	< 0.001	–	<0.001	–	< 0.001

Model 1: unadjusted; Model 2: adjusted for gender, age, race/ethnicity, poverty income ratio and education level; Model 3: adjusted for gender, age, race/ethnicity, poverty income ratio, education level, estimated glomerular filtration rate (eGFR), albumin, alanine aminotransferase (ALT), aspartate aminotransferase (AST), total cholesterol, alcohol use, smoking status, physical activity, sedentary behavior and medical history of cancer, coronary heart disease, hypertension, diabetes, stroke. UHR: uric acid to high-density lipoprotein cholesterol ratio; OR: odds ratio; CI: confidence interval.

### The mediating role of oxidative stress factor (total bilirubin) and systemic inflammation markers (SII, PLR, NLR) in the UHR-sarcopenia association

**FIGURE 2:**
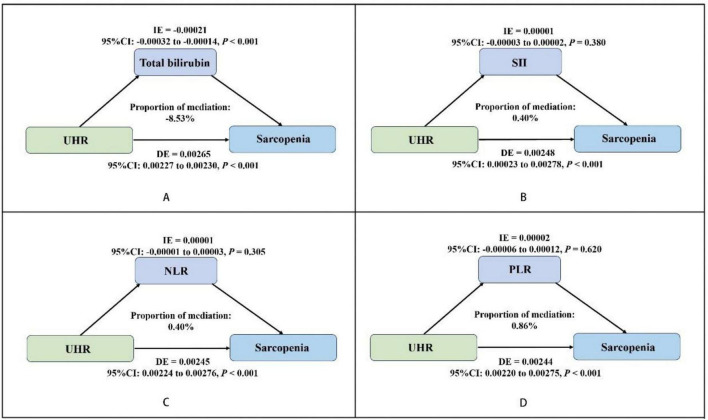
Estimated proportion of the association between UHR and sarcopenia mediated by oxidative stress factor (total bilirubin) **(A)**, and systemic inflammation markers (SII, NLR, PLR) **(B–D)**. Adjusted for gender, age, race/ethnicity, poverty income ratio, education level, estimated glomerular filtration rate (eGFR), albumin, alanine aminotransferase (ALT), aspartate aminotransferase (AST), total cholesterol, alcohol use, smoking status, physical activity, sedentary behavior and medical history of cancer, coronary heart disease, hypertension, diabetes, stroke. UHR, uric acid to high-density lipoprotein cholesterol ratio; SII, systemic immune-inflammation index; NLR, neutrophil-to-lymphocyte ratio; PLR, platelet-to-lymphocyte ratio; IE, indirect effect; DE, direct effect; OR, odds ratio; CI, confidence interval.

We conducted a parallel mediation analysis to investigate the potential mediating role of oxidative stress factor (total bilirubin) and systemic inflammation markers (SII, PLR, NLR). The results showed that the total effect (TE) and direct effect (DE) of UHR on sarcopenia were 0.00244 and 0.00265, respectively (*P* < 0.001). The indirect effect (IE) of UHR on sarcopenia through bilirubin was −0.00021 (*P* < 0.001), with the proportion mediated by bilirubin being −8.53% (Figure 2A). The mediation effect of systemic inflammation markers (SII, PLR, NLR) on the UHR-sarcopenia association was not statistically significant (Figures 2B–D).

### Subgroup analysis and interaction tests

To further investigate the impact of the positive association between UHR and sarcopenia in different populations, subgroup analysis and interaction tests were performed based on gender, age, physical activity, and sedentary behavior. The results demonstrated a borderline significant age-specific interaction (*P* for interaction = 0.048), suggesting that the association between UHR and sarcopenia may vary by age group, with a stronger association observed in participants aged < 40 years (OR = 1.078; 95% CI 1.053–1.103; *P* < 0.001) compared to those ≥ 40 years (OR = 1.041; 95% CI 1.013–1.069; *P* = 0.005). No significant interaction effects were found for sex, physical activity, or sedentary time (all *P* for interaction > 0.05). In the stratified analyses, UHR showed significant positive associations with sarcopenia in both sexes (men: OR = 1.06, *P* < 0.001; women: OR = 1.051, *P* = 0.010). For physical activity levels, the association reached statistical significance only in the most active group (≥ 300 min/week: OR = 1.081, *P* < 0.001). Regarding sedentary behavior, significant associations were observed for both 180–299 min/day (OR = 1.101, *P* < 0.001) and ≥ 420 min/day (OR = 1.042, *P* = 0.003) groups (Table 3).

**TABLE 3:** Subgroup analysis of the association of UHR and sarcopenia.

Subgroup	OR (95% CI)	*P*-value	*P*-value for interaction
Gender			0.715
Male	1.06 (1.033, 1.088)	< 0.001	–
Female	1.051 (1.012, 1.092)	0.010	–
Age strata			0.048
< 40	1.078 (1.053, 1.103)	< 0.001	–
≥ 40	1.041 (1.013, 1.069)	0.005	–
Physical activity, *n* (%)			0.249
0 min/week	1.032 (0.991, 1.074)	0.124	–
1–149 min/week	1.005 (0.949, 1.063)	0.872	–
150–299 min/week	1.043 (0.966, 1.127)	0.274	–
≥ 300 min/week	1.081 (1.051, 1.112)	< 0.001	–
Sedentary behavior, *n* (%)			0.231
0–179 min/d	1.046 (0.997, 1.098)	0.063	–
180–299 min/d	1.101 (1.059, 1.145)	< 0.001	–
300–419 min/d	1.04 (0.988, 1.095)	0.131	–
≥ 420 min/d	1.042 (1.014, 1.07)	0.003	–

### Non-linear relationship between UHR and sarcopenia

**FIGURE 3:**
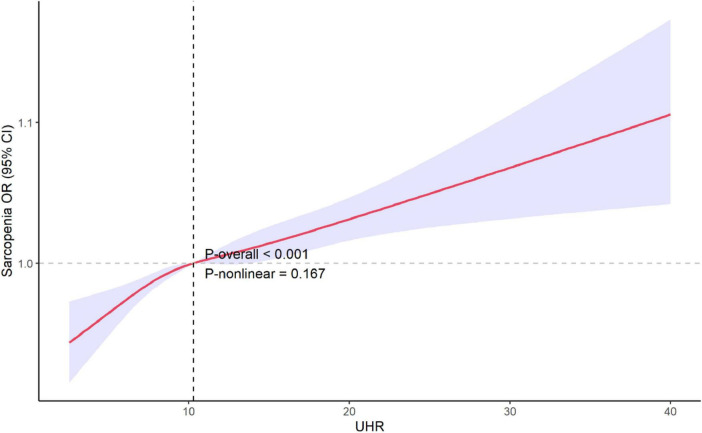
Restricted cubic spline explores the potential non-linear relationships between UHR and sarcopenia. The 95% confidence levels (shaded areas) were adjusted for gender, age, race/ethnicity, poverty income ratio, education level, estimated glomerular filtration rate (eGFR), albumin, alanine aminotransferase (ALT), aspartate aminotransferase (AST), total cholesterol, alcohol use, smoking status, physical activity, sedentary activity and medical history of cancer, coronary heart disease, hypertension, diabetes, stroke. UHR, uric acid to high-density lipoprotein cholesterol ratio; OR, odds ratio; CI, confidence interval.

Restricted cubic spline was used to explore the potential non-linear relationship between UHR and the risk of sarcopenia. The results, as shown in Figure 3, revealed no significant non-linear relationship between UHR and sarcopenia after adjusting for all covariates (*P* = 0.167).

### UHR and all-cause mortality in sarcopenia patients

During a median follow-up of 63 months, 25 participants with missing mortality data were excluded, leaving 166 deaths (mortality rate 1.61%) among the 10,308 participants. Weighted multivariate Cox proportional hazards regression analysis was performed to assess the association between UHR and all-cause mortality in sarcopenia patients. The results showed that without adjusting for covariates (Model 1), UHR was significantly positively associated with all-cause mortality (HR = 1.053; 95% CI: 1.024–1.083; *P* < 0.001), meaning that for each 1% increase in UHR, the risk of all-cause mortality in sarcopenia patients increased by approximately 5.3%. After adjusting for gender, age, race, education level, and PIR (Model 2), the association between UHR and mortality showed marginal statistical significance (HR = 1.034; 95% CI: 0.999–1.070; *P* = 0.055). After adjusting for all covariates (Model 3), UHR maintained a positive association with all-cause mortality in sarcopenia patients (HR = 1.023; 95% CI: 0.990–1.056), though this association did not reach statistical significance (*P* = 0.173) (Table 4).

**TABLE 4:** Weighted multivariate Cox proportional hazards regression analysis of the association between UHR and all-cause mortality in sarcopenia patients.

Variables	Model 1		Model 2		Model 3	
	**HR (95% CI)**	***P*-value**	**HR (95% CI)**	***P*-value**	**HR (95% CI)**	***P*-value**
UHR	1.053 (1.024, 1.083)	< 0.001	1.034 (0.999, 1.070)	0.055	1.023 (0.990, 1.056)	0.173
**Categories**						
Q 1	Reference	–	Reference	–	Reference	–
Q 2	0.848 (0.474, 1.518)	0.579	0.762 (0.429, 1.355)	0.355	0.802 (0.451, 1.424)	0.451
Q 3	1.857 (1.126, 3.064)	0.015	1.470 (0.808, 2.672)	0.207	1.281 (0.690, 2.378)	0.433
Q 4	2.041 (1.126, 3.700)	0.019	1.414 (0.702, 2.848)	0.332	1.296 (0.635, 2.646)	0.477
*P* for trend	–	0.004	–	0.165	–	0.303

Model 1: unadjusted; Model 2: adjusted for gender, age, race/ethnicity, poverty income ratio and education level; Model 3: adjusted for gender, age, race/ethnicity, poverty income ratio, education level, estimated glomerular filtration rate (eGFR), albumin, alanine aminotransferase (ALT), aspartate aminotransferase (AST), total cholesterol, alcohol use, smoking status, physical activity, sedentary behavior and medical history of cancer, coronary heart disease, hypertension, diabetes, stroke. UHR, uric acid to high-density lipoprotein cholesterol ratio; HR, hazard ratio; CI, confidence interval.

## Discussion

The results of this study indicate a significant positive association between the UHR and the risk of sarcopenia. Through mediation analysis, we further confirmed the mediating role of oxidative stress in this relationship, revealing that UHR may promote the development of sarcopenia via oxidative stress mechanisms. Additionally, our study showed an association between UHR and all-cause mortality in sarcopenia patients, suggesting that UHR could be an important predictive factor for the prognosis of sarcopenia. This finding provides new insights into the potential of UHR as a risk factor for sarcopenia, warranting further exploration and validation in future studies. Notably, this is the first study to investigate UHR as a risk factor for sarcopenia, offering new directions for early identification and intervention.

Uric acid is the primary product of purine metabolism in the body (35), and elevated levels are associated not only with various metabolic diseases (36). Still, they can also directly affect muscle tissue through multiple biological pathways (13, 37–39). Studies have shown that increased uric acid production activates xanthine oxidoreductase (XOR), generating reactive oxygen species (ROS) and inducing oxidative damage (37). Simultaneously, uric acid inhibits the insulin receptor substrate 1-protein kinase B (IRS1-Akt) signaling pathway, reducing insulin-mediated glucose uptake and contributing to skeletal muscle insulin resistance, disrupting energy metabolism (38). Furthermore, uric acid impairs mitochondrial function by reducing oxidative phosphorylation and electron transport chain efficiency, compromising ATP production and weakening muscle contraction and repair capacity (39). It also promotes intracellular triglyceride accumulation in muscle cells and upregulates the expression of transforming growth factor-beta 1 (TGF-β1) and nicotinamide adenine dinucleotide phosphate oxidase 4 (NOX4), exacerbating oxidative stress and inflammatory responses, which further disrupt muscle structure and function (40). Collectively, these changes lead to muscle fiber atrophy and diminished strength, driving the onset and progression of sarcopenia (13). HDL-C also plays a critical role in maintaining muscle health. HDL-C can directly scavenge mitochondrial ROS, effectively protecting muscle fibers from oxidative damage (41). Normally functioning HDL-C promotes reverse cholesterol transport in skeletal muscle cells to maintain membrane stability and enhances glucose uptake by increasing Akt phosphorylation in the insulin signaling pathway (42). When HDL-C levels are reduced or its function is impaired, the body may activate the ubiquitin-proteasome system and autophagy pathways (43–45), weakening these protective effects and leading to increased muscle protein degradation and diminished fiber regeneration. Additionally, reduced HDL-C suppresses the insulin-like growth factor-1/protein kinase B (IGF-1/Akt) pathway, impairing satellite cell proliferation and differentiation while promoting fibrosis, ultimately resulting in impaired muscle regeneration and atrophy (42). Elevated uric acid levels and/or reduced or dysfunctional HDL-C contribute to an increased UHR. As a composite biomarker, an elevated UHR reflects a systemic imbalance between pro-oxidant/pro-inflammatory and antioxidant/anti-inflammatory homeostasis (21, 46). This imbalance accelerates muscle loss through the aforementioned synergistic mechanisms, ultimately promoting the development and progression of sarcopenia.

Our study further revealed, through mediation analysis, that total bilirubin plays a negative mediating role in the relationship between UHR and sarcopenia, with a proportion mediated of −8.51%. The underlying mechanisms may involve that elevated UHR, on one hand, excessively activates NADPH oxidase (NOX) to generate ROS, thereby activating the nuclear factor erythroid 2-related factor 2 (Nrf2)/heme oxygenase-1 (HO-1) signaling pathway and promoting heme degradation into bilirubin (37, 40, 47). Simultaneously, elevated UHR suppresses the adenosine 5’-monophosphate-activated protein kinase (AMPK) signaling pathway, indirectly downregulating the expression of the bilirubin transporter multidrug resistance-associated protein 2 (MRP2) and reducing bilirubin excretion, leading to its systemic accumulation (47). Elevated bilirubin, as a potent endogenous antioxidant, mitigates UHR-induced sarcopenia progression through multifaceted mechanisms including direct ROS scavenging, AMPK signaling pathway-dependent activation of the Nrf2 antioxidant system, suppression of NF-κB-mediated inflammatory responses, and enhancement of mitochondrial function (9, 47). These findings not only align with previous research supporting the protective role of bilirubin in sarcopenia (12) but also provide novel molecular-level evidence for bilirubin as a protective mediator linking metabolic dysregulation (UHR) to sarcopenia, reinforcing the “metabolic disturbance-oxidative stress-muscle loss” pathophysiological axis (5, 48). Although these results require validation through longitudinal studies, they suggest potential therapeutic targets (e.g., enhancing antioxidant capacity) for sarcopenia prevention and treatment in patients with metabolic abnormalities. Although previous studies have suggested systemic inflammation as a potential pathological mechanism in sarcopenia development (4, 5), our study did not identify significant mediating effects of SII, PLR, or NLR in the UHR-sarcopenia association. This discrepancy may stem from fundamental differences in inflammatory dynamics: while these peripheral blood cell ratios primarily reflect acute-phase inflammatory responses, sarcopenia pathogenesis likely involves chronic low-grade inflammation mediated by sustained cytokine activity (e.g., IL-6, TNF-α) (49). Furthermore, the pathological profile of individuals with elevated UHR appears more consistent with an “oxidative stress-dominant” paradigm, where compensatory activation of antioxidant systems may take pathological precedence over systemic inflammatory responses (5).

Further analyses revealed a positive trend between elevated UHR and all-cause mortality in sarcopenia patients, though this association did not reach statistical significance after multivariable adjustment (*P* > 0.05), suggesting the need for larger-scale validation of its predictive value. Subgroup analyses demonstrated a more pronounced UHR-sarcopenia association in the younger population (< 40 years; *P* for interaction = 0.048). However, given the borderline significance and absence of significant differences in other subgroups, these findings should be interpreted with caution. Further validation in independent cohorts and mechanistic investigations are warranted.

### Strengths and limitations

Our study has several strengths. First, it is the first to explore the relationship between UHR and the occurrence of sarcopenia, and to examine oxidative stress factors and systemic inflammation markers as mediators. Additionally, this study investigates the association between UHR and all-cause mortality in sarcopenia patients, allowing for a more comprehensive understanding of UHR’s potential role in the development and clinical prognosis of sarcopenia. Third, our study is based on a large sample of United States adult population data, ensuring external validity and generalizability of the results.

However, there are several limitations in this study. First, it is a cross-sectional study, meaning that we cannot establish causality between UHR and the occurrence of sarcopenia. Therefore, further prospective studies are needed to confirm the relationship between these two factors. Second, although we adjusted for multiple potential confounders (including major metabolic disorders such as hypertension, diabetes, coronary heart disease, and stroke), other metabolic conditions like gout were not accounted for. Third, due to substantial missing data on medication use in NHANES (particularly urate-lowering and lipid-modifying drugs), we were unable to adjust for pharmacological factors that may influence uric acid and HDL-C levels. These unmeasured confounders might have affected our findings.

## Conclusion

Our findings suggest that elevated UHR is significantly associated with sarcopenia prevalence and shows a trend toward association with all-cause mortality in this population. While these cross-sectional observations indicate UHR may serve as a potential marker for sarcopenia risk assessment, the predictive value of UHR for sarcopenia incidence and mortality outcomes requires further validation in longitudinal studies. Future prospective studies are needed to establish the temporal relationship and potential causal mechanisms between UHR and sarcopenia development.

## Data Availability

The original contributions presented in this study are included in this article/supplementary material, further inquiries can be directed to the corresponding author.

## Footnotes

1
https://www.cdc.gov/nchs/nhanes
